# GTSE1 Is a Microtubule Plus-End Tracking Protein That Regulates EB1-Dependent Cell Migration

**DOI:** 10.1371/journal.pone.0051259

**Published:** 2012-12-07

**Authors:** Massimilano Scolz, Per O. Widlund, Silvano Piazza, Debora Rosa Bublik, Simone Reber, Leticia Y. Peche, Yari Ciani, Nina Hubner, Mayumi Isokane, Martin Monte, Jan Ellenberg, Anthony A. Hyman, Claudio Schneider, Alexander W. Bird

**Affiliations:** 1 Laboratorio Nazionale The Interuniversity Consortium for Biotechnology, Area Science Park, Trieste, Italy; 2 Max Planck Institute of Molecular Cell Biology and Genetics, Dresden, Germany; 3 Department of Molecular Cancer Research, Universitair Medisch Centrum Utrecht, Utrecht, The Netherlands; 4 European Molecular Biology Laboratory, Cell Biology and Biophysics Unit, Heidelberg, Germany; 5 Department of Medical and Biological Sciences, University of Udine, Udine, Italy; University of Edinburgh, United Kingdom

## Abstract

The regulation of cell migration is a highly complex process that is often compromised when cancer cells become metastatic. The microtubule cytoskeleton is necessary for cell migration, but how microtubules and microtubule-associated proteins regulate multiple pathways promoting cell migration remains unclear. Microtubule plus-end binding proteins (+TIPs) are emerging as important players in many cellular functions, including cell migration. Here we identify a +TIP, GTSE1, that promotes cell migration. GTSE1 accumulates at growing microtubule plus ends through interaction with the EB1+TIP. The EB1-dependent +TIP activity of GTSE1 is required for cell migration, as well as for microtubule-dependent disassembly of focal adhesions. GTSE1 protein levels determine the migratory capacity of both nontransformed and breast cancer cell lines. In breast cancers, increased GTSE1 expression correlates with invasive potential, tumor stage, and time to distant metastasis, suggesting that misregulation of GTSE1 expression could be associated with increased invasive potential.

## Introduction

Cancer cells that have become metastatic are defined by an increased motility, and the modulation of molecular pathways controlling cell migration is important for the progression to metastasis [1]–[4]. These pathways are normally regulated so that migration occurs only at specific times within the cell-cycle, or in response to distinct environmental cues.

Several regulatory pathways controlling cell migration are dependent on the microtubule cytoskeleton, as well as the dynamic regulation of microtubule-associated proteins (MAPs) [5]. MAPs are frequently found overexpressed in tumors, where they are thought to promote cancer progression and resistance to MT-targeting chemotherapy drugs in part by altering microtubule dynamics and stability and promoting chromosomal instability in mitosis [6]–[7]. Due to their important role in cell motility, misregulation of microtubule functions may also potentially contribute to cancer progression through misregulation of cell migration, by promoting tumor cell invasion and metastasis [5], although there is less evidence for this to date. Furthermore, microtubule-targeting drugs such as paclitaxel have proven effective at treating cancers, but the pathways through which they work remain unclear [8]. To better understand how regulation of microtubule-dependent cell migration affects cancer progression, it is necessary to elucidate the contributions of individual MAPs.

Several microtubule-dependent pathways controlling cell migration act by ultimately stimulating actin polymerization at the leading edge of a migrating cell [5]. At the same time, microtubules promote cell migration through the modulation of dynamic cell adhesion properties, by stimulating both the stabilization and disassembly of focal adhesion complexes (FAs) [9]. FAs are large macromolecular complexes required for cell adhesion to the extracellular matrix, as well as signaling from the ECM to the cell. FAs are dynamic, and both their assembly and disassembly are regulated by multiple factors [10], [11]. As they migrate, cells must form new FAs at their leading edge, and then disassemble these FAs as they move towards the middle and rear of the cell. The disassembly of FAs appears to be induced when microtubules grow towards and touch (“target”) focal adhesions, suggesting MAPs are also critical in this process [12]–[14].

One subclass of MAPs that is ideally positioned to regulate microtubule dynamics and interactions within cells, due to its members’ localization at the plus end of microtubules, is +TIPs (microtubule plus-end tracking proteins) [15]–[16]. +TIPs generally refer to proteins that localize to growing MT ends, but do not necessarily associate with microtubules themselves. Many of the +TIP proteins identified to date actually localize to the growing microtubule end by virtue of an interaction with the EB (end-binding) family of +TIP proteins. EB proteins associate directly with MTs through a N-terminal calponin homology domain [17]. EB1-interacting +TIP proteins bind to EB1 via conserved domains located in the C-terminal region of EB1 [18], [19]. The majority of these bind EB1 specifically via short interaction motifs residing in basic and serine-rich regions, named “SKIP” (or “SxIP”) motifs, for the original defined consensus sequence [20]. EB1 thus serves as an essential hub of localization for many +TIPs, and is ideally situated to play key roles in regulation of +TIP protein localization and activities. Indeed, recent studies have provided examples of phosphorylation of +TIPs affecting their interaction with EB1 and localization to growing microtubule ends [18], [20]–[22].

Among several functions, EB1 is required for cell migration [23]–[25]. Because of EB1’s ability to recruit likely dozens of different +TIPs to microtubules in a complex fashion [26], it is difficult to study individual mechanisms through EB1 perturbation alone, thus requiring the analysis of individual EB1-interacting +TIPs and their unique regulation. A few EB1-interacting +TIPs (i.e. APC, ACF7, CLASP1/2, and CLIP170) have been shown to play roles in migration-promoting pathways [22], [23], [27]–[30]. Most evidence defines how they work together at the leading edge of a migrating cell to promote stabilization of microtubules, cell adhesion, microtubule attachment at the cortex, and stimulation of actin polymerization [5], [9]. How EB1 affects additional pathways promoting cell migration, such as focal adhesion disassembly, is less clear, although the EB1-interacting +TIPs ACF7 and CLIP-170 have been shown to be important for this activity [28], [30], [31].

We previously identified the protein GTSE1 (G-2 and S-phase expressed 1) as a negative regulator of p53 that can shuttle between the cytoplasm and nucleus. After DNA damage, GTSE1 accumulates the nucleus, where it interacts with p53 and shuttles it out of the nucleus to promote its downregulation and recovery from the p53-induced G2 DNA damage checkpoint [32] [33], [34]. In the absence of DNA damage, GTSE1 localizes to interphase MT networks [35]–[37], and has also been found associated with clathrin-containing complexes [38], [39], but the function of GTSE1 at microtubules has not been elucidated.

Here we have identified GTSE1 as a microtubule-associated +TIP protein required for EB1-dependent cell migration. GTSE1 interacts directly with microtubules in interphase, and is enriched at growing microtubule plus ends through interaction with EB1. We have found a positive relationship between GTSE1 protein levels and a cell’s migratory capacity. Focal adhesion turnover activity is also dependent on GTSE1, suggesting that GTSE1’s affect on cell migration is mediated through stimulation of microtubule-dependent focal adhesion disassembly. Furthermore, the impact of GTSE1 on both cell migration and focal adhesion turnover is dependent on its interaction with EB1 and tip-tracking activity. Combined with evidence that GTSE1 expression levels correlate with tumor invasiveness and metastasis in breast cancer, these results point to the possibility that misregulation of the +TIP activity of GTSE1 may promote pathways supporting metastasis, through upregulation of FA disassembly leading to loss of adhesion and increased cell motility.

## Results

### GTSE1 is an EB1-dependent Microtubule Plus End Growth Tracking Protein

**Figure 1:**
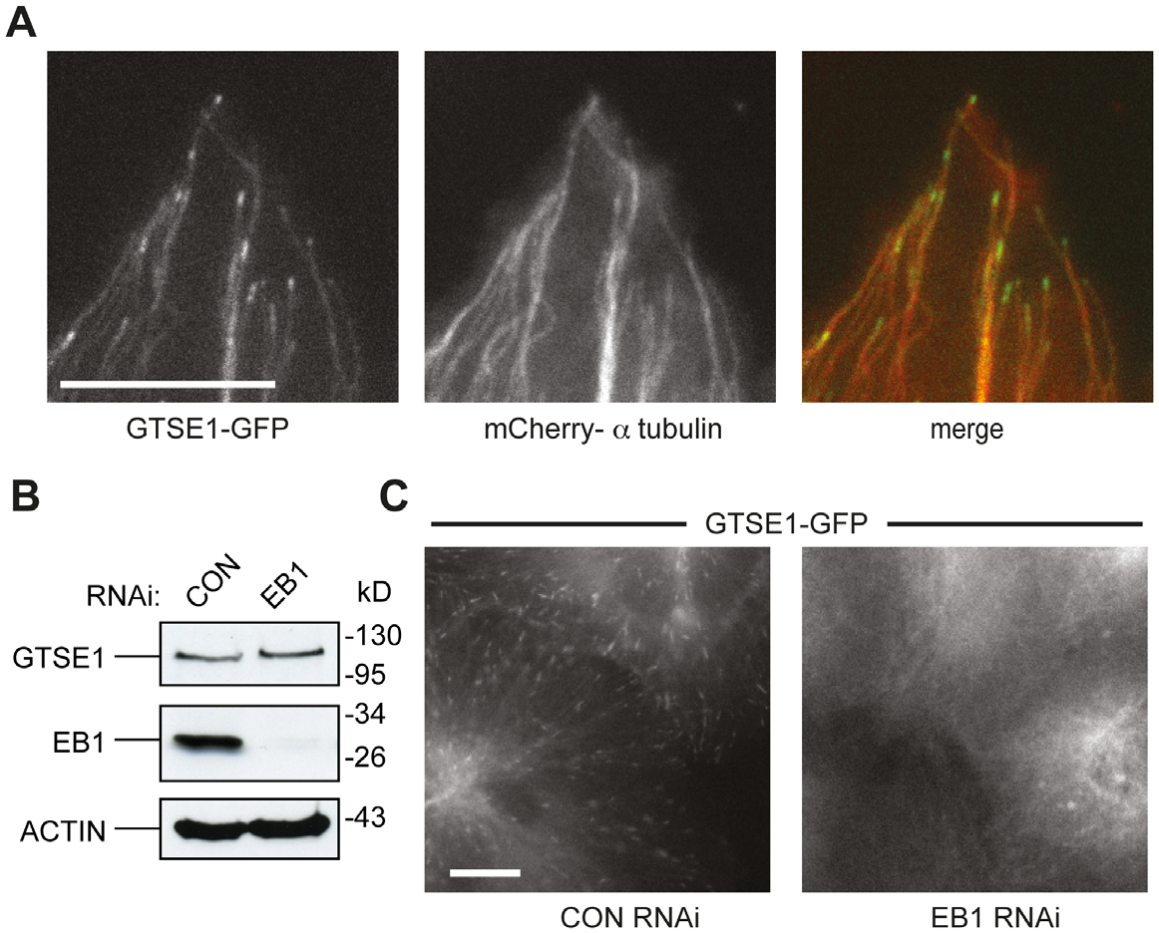
GTSE1 is an EB1-dependent microtubule plus end growth tracking protein. (A) Still images of a live U2OS cell stably expressing GTSE1-GFP and mCherry-alpha-tubulin from Movie S1. GTSE1-GFP is enriched at growing microtubule plus ends, and also associated with the microtubule lattice. (B) Western blot showing GTSE1 and EB1 levels after EB1 RNAi. U2OS cells stably expressing GFP-GTSE1 were transfected with a control (siCONT) or EB1 (siEB1) siRNA for 36 h. Blots were probed with antibodies against GFP, EB1 or actin (loading control). (C) Still images of live U2OS cells expressing GTSE1-GFP after transfection with control (CON) or EB1 siRNA, from Movies S2 and S3. After EB1 depletion, GTSE1-GFP no longer tracks growing microtubule ends, but remains associated with the microtubule lattice.

We previously described GTSE1 as localizing coincident with interphase microtubule networks in antibody stainings [35]–[37], and in a yeast 2-hyrid screen for GTSE1-interacting proteins, EB1 emerged as a strong candidate. To investigate more closely potential microtubule- and EB1- related functions of GTSE1, we tagged the *GTSE1* gene with a C-terminal GFP tag within a bacterial artificial chromosome (BAC), and transfected U2OS cells and mouse embryonic stem cells (R1/E) with this construct. Time lapse imaging of stably transfected cells revealed that in addition to localizing to microtubules, GTSE1-GFP was enriched on what appeared to be growing microtubule tips in interphase in both cell types (Figure 1A,C; Movie S1,S2,S3). Live analysis of GTSE1-GFP cells stably co-transfected with alpha-tubulin-mCherry confirmed that GTSE1-GFP accumulates at growing microtubule tips (Figure 1A, Movie S1), identifying GTSE1 as a +TIP.

To determine if GTSE1 required EB1 for its localization to growing MT ends, we depleted U2OS GTSE1-GFP cells of EB1 by RNAi and recorded short time-lapse movies (Movie S3,S4). RNAi depletion of EB1 efficiently depleted EB1 protein, but did not affect the expression levels of GTSE1 (Figure 1B). GTSE1-GFP no longer tracked microtubule tips after EB1 depletion, but still associated with the microtubule lattice, confirming that GTSE1 is dependent on EB1 for +TIP localization (Figure 1C).

### GTSE1 is Recruited to Microtubule Plus Ends Through Short EB1-interaction Motifs

**Figure 2:**
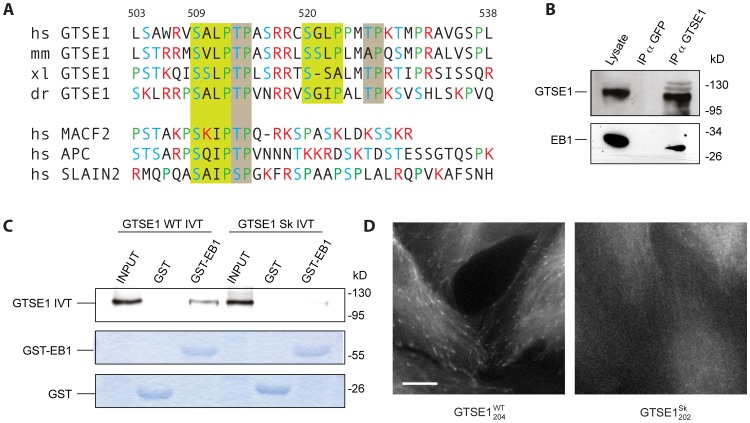
GTSE1 is recruited to microtubule plus ends through short EB1-interaction motifs. (A) Sequence alignment of hGTSE1 amino acids 503–538 that contain tandem conserved SKIP-like motifs. The first four rows contain GTSE1 homologs from human (hs), mouse (mm), Xenopus (xl) and zebrafish (dr). The last three rows show conserved regions from other human +TIPs. SKIP-like motifs are highlighted in green boxes. Conserved TP motifs are highlighted in grey boxes. Basic residues are colored red, serines and threonines are colored blue. (B) GTSE1 immunoprecipitates EB1 in U2OS cells. U2OS cell lysates were immunoprecipitated with anti-GTSE1 antibody, or anti-GFP as a control. Input lysate and immunoprecipitated fractions were run by SDS-PAGE and Western blotted with either anti-GTSE1 or anti-EB1 antibody. (C) *In vitro* pull-down binding assay using purified GST or GST-EB1 fusion proteins incubated with *in vitro* translated ^35^S-labeled GTSE1 WT (hGTSE1 WT IVT) or GTSE1 mutated at the SKIP motifs (L511N P512N L522N P523N) (GTSE1 Sk IVT). Inputs represent 20% of IVTs used for pull-down assays. The top gel shows IVT GTSE1 by autoradiograph, bottom gels are commassie stained. GST-EB1 interacts with *in vitro* translated GTSE1, but not GTSE1 mutated at SKIP motifs. (D) Still images of live clonal U2OS cells expressing wild type GTSE1-GFP (GTSE1^WT^_204_) or GTSE1-GFP mutated at the SKIP motifs (L511N P512N L522N P523N) (GTSE1^Sk^_202_)(Movie S4). Similar to EB1 depletion, the mutated GTSE1-GFP does not track growing microtubule tips, but localizes to the microtubule lattice. All scale bars represent 10 microns.

Analysis of the GTSE1 sequence and secondary structure prediction revealed that GTSE1 is a mostly intrinsically disordered protein (IDP), with the exception of a short N-terminal region of 100 amino acids predicted to fold into an ordered secondary structure. Within the disordered regions are multiple potential “SKIP”-like EB1-interaction motifs surrounded by basic residues, including two highly conserved tandem motifs (residues 509–512 and 520–523; here SxLP; Figure 2A), which suggested a direct interaction with and +TIP targeting via EB1. To determine if EB1 and GTSE1 interacted, we immunoprecipitated endogenous GTSE1 from U2OS cells and probed for EB1. Anti-GTSE1 antibody, but not a control antibody (anti-GFP), efficiently co-immunoprecipitated endogenous EB1 (Figure 2B).

Proteins that interact with EB1 through the SKIP motif generally bind to the EBH (EB-homology) domain located in the C-terminus of EB1 [20]. We investigated which region of EB1 was required for interaction with GTSE1 by performing coimmunoprecipitations with cells transiently transfected with GTSE1-HA and different EB1-GFP fragment constructs. Ectopically expressed GTSE1-HA efficiently coimmunoprecipitated full length EB1-GFP (Figure S1A,B). A C-terminal EB1-GFP fragment containing the EBH domain (residues 143–268) was also immunoprecipiated with GTSE1-HA, but an N-terminal EB1-GFP fragment (residues 1–143) was not (Figure S1B), suggesting the EB1 EBH domain was also required for GTSE1 interaction.

To test if we could detect this interaction *in vitro*, we performed an *in vitro* pull-down binding assay using a recombinant purified GST-EB1 fusion protein incubated with *in vitro* translated ^35^S-labeled GTSE1. GST-EB1, but not GST alone, was able to pull down hGTSE1, confirming an interaction (Figure 2C). The reciprocal experiment, using recombinant GST-GTSE1 and ^35^S-in vitro translated EB1 further verified this result (Figure S1C). ^35^S-labeled GTSE1 from mouse and *Xenopus laevis* was also pulled down by GST-EB1, further indicating that this interaction is conserved (Figure S1D).

To determine if the interaction of GTSE1 with EB1 was mediated through the conserved SKIP-like motifs, we mutated the two leucine and proline residues within these motifs known to be critical for the EB1 interaction in other proteins (SALP to SANN and SGLP to SGNN; [20]), and again tested for pull down by GST-EB1 (Figure 2C). These mutations indeed abolished the interaction of GTSE1 with EB1. To test if these residues were also critical in cells for the tip-tracking activity of GTSE1, we used counterselection recombineering [40] to engineer the same mutations into a *GTSE1-*GFP BAC transgene (“GTSE1^Sk^”). Similar to EB1 depletion, mutation of tandem SxLP motifs in GTSE1-GFP abolished interphase GTSE1-GFP tip tracking, but not microtubule lattice association (Figure 2D, Movie S5,S6). Together, these results identify GTSE1 as a +TIP that is recruited to growing microtubule tips by interaction with EB1. A recent screen for novel +TIPs containing SKIP-like motifs also identified GTSE1 as one of 20 uncharacterized EB1-interacting proteins [41].

### GTSE1 Binds Directly to the Microtubule Lattice Independent of EB1

**Figure 3:**
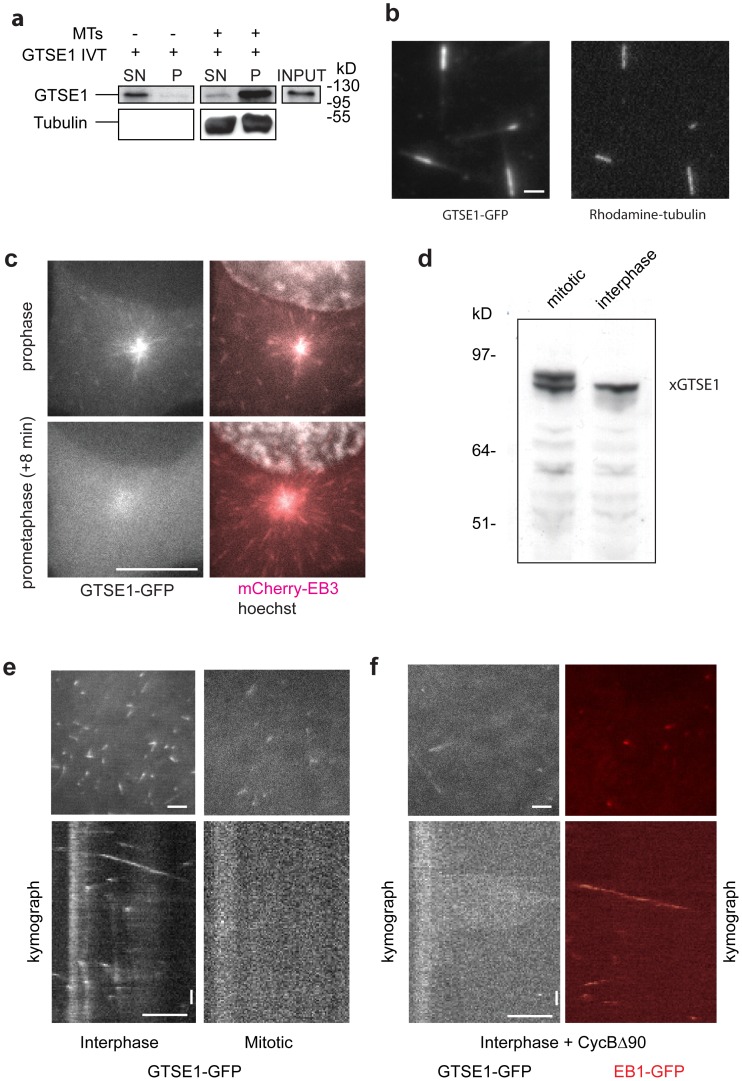
GTSE1 binds directly to the microtubule lattice and stops tip tracking in mitosis. (A) Microtubule co-sedimentation assay. *In vitro* translated ^35^S-labeled GTSE1 (GTSE1 IVT) was incubated with taxol-stabilized microtubules (MTs +) or with taxol-containing buffer (without microtubules; MTs −). Supernatant (SN) and pellet (P) fractions were separated by SDS-PAGE and the presence of GTSE1 in each fraction was detected by autoradiography. (B) Still images of a TIRF field showing GTSE1-GFP (left panel) binding to rhodamine-labeled GMPCPP seeds (right panel) and along the growing MT lattice. Scale bar represents 2 microns. (C) Still images of live U2OS cells stably expressing GTSE1-GFP and EB3-mCherry with DNA labeled with Hoechst, from Movie S5. Time GTSE1-GFP tracks growing plus ends in prophase, but not 8 minutes later in prometaphase. Scale bar represents 10 microns. (D) Western blot of endogenous GTSE1 in Xenopus meiotic (M) and interphase (I) extract. (E) Still images of TIRF fields and representative kymographs of GMPCPP-stabilized microtubules incubated with interphase (Movie S7) or mitotic (Movie S8) *Xenopus* egg extracts and purified hGTSE1-GFP protein. Horizontal scale bar represents 5 microns, vertical scale bar 10 seconds. (F) Still images of TIRF fields and representative kymographs of GMPCPP-stabilized microtubules incubated with mitotic *Xenopus* egg extracts and purified hGTSE1-GFP or EB1-GFP protein, after addition of purified cyclinBΔ90, from Movies S9 and S10.

During interphase, GTSE1 not only tracks growing microtubules, but also associates with the MT lattice, in an EB1-independent manner (Figure 1A,C; Figure 2D). To investigate whether GTSE1 interacted with the microtubule lattice directly, or was dependent on other factors, as is the tip localization, we first asked if microtubules could pull down GTSE1 protein in a microtubule co-sedimentation assay. *In vitro*-translated 35S-labeled GTSE1 was incubated with taxol-stabilized microtubules or with taxol and buffer alone (Figure 3A). With buffer alone, the majority GTSE1 protein remained in the supernatant, while with the addition of microtubules, the majority of GTSE1 protein was pelleted along with the microtubules, consistent with an interaction of GTSE1 with microtubules.

To confirm the ability of GTSE1 to bind microtubules, we assayed for microtubule binding of purified recombinant hGTSE1-GFP by total-internal-reflection fluorescence (TIRF) microscopy. Purified hGTSE1-GFP and unlabeled tubulin were added to rhodamine-labeled, GMPCPP stabilized microtubule seeds. hGTSE1-GFP associated with the dynamic microtubule lattice, and was highly enriched along the microtubule seeds, confirming a direct interaction (Figure 3B, Movie S7). Therefore the EB1-independent microtubule lattice localization of GTSE1 in cells is most likely due to a direct interaction.

### GTSE1+TIP Activity is Lost during Mitosis and Regulated by Phosphorylation

Initial imaging of mitotic cells expressing GTSE1-GFP showed no signs of microtubule tip-tracking [38]. To observe precisely when GTSE1+TIP localization changes during mitosis, we filmed stable cell lines expressing both GTSE1-GFP and EB3-mCherry, which tracks microtubule plus-ends throughout the cell cycle. Short-term time lapse movies of GTSE1-GFP mCherry-EB3 cells stained with Hoechst (DNA) entering and exiting mitosis revealed that while EB3 tip-tracking is consistent throughout, GTSE1 tip-tracking stops at the prophase to prometaphase transition, approximately concurrent with nuclear envelope breakdown, and resumes coincident with anaphase onset (Figure 3C, Movie S7,S8).

We previously reported that murine Gtse1 is hyperphosphorylated in mitosis in NIH3T3 cells [35], suggesting that the +TIP activity of GTSE1 may be negatively regulated my phosphorylation. In order to closely analyze the tip-tracking ability of GTSE1 in defined cell cycle stages, we developed a system whereby interphase or mitotic *Xenopus* extracts are flowed over purified stabilized microtubules to observe microtubule dynamics by TIRF microscopy (see methods). As in mammalian cells, endogenous *Xenopus* gtse1 was hyperphosphorylated in mitotic extracts, suggesting a similar regulation in *Xenopus* (Figure 3D). Adding purified hGTSE1-GFP to interphase *Xenopus* extracts resulted in dramatic tip-tracking behavior of the purified protein, in addition to microtubule lattice association (Figure 3E; Movie S10). In contrast, we did not observe any tip-tracking events when purified hGTSE1-GFP was added to a mitotic extract (Figure 3E, Movie S11). Purified hEB1-GFP maintained robust tip tracking in both interphase and mitotic extracts under the same experimental conditions (data not shown). Together these results confirmed that as in mammalian cells, in *Xenopus* extracts GTSE1 is an interphase-specific microtubule tip-tracking protein.

Recent studies have shown that the interaction between +TIP proteins and EB1 can be regulated through phosphorylation of sites located around SKIP motifs [18], [20]–[22]. Notably, GTSE1 contains conserved putative CDK1 (Cyclin Dependent Kinase 1) phosphorylation sites (TP) located directly adjacent to both SKIP-like motifs (Figure 2A). Furthermore, a cell-cycle-dependent phosphoproteome analysis in human cells identified GTSE1 peptides containing these TP sites as hyperphosphorylated in mitosis (www.phosida.com; [42], [43]). We independently identified these specific residues as mitotic phosphorylation sites by mass spectrometry as well (Table S1). To investigate if CDK1 activity was responsible for negatively regulating the tip-tracking ability of GTSE1, we performed the same assays for tip tracking as described above in interphase *Xenopus* extracts, but after stimulation of CDK1 activity. CDK1 activity is low in interphase extracts, but can be stimulated by the addition of purified non-degradable cyclinB (cyclinBΔ90) [44], [45]. We found that shortly after addition of cyclinBΔ90 and hGTSE1-GFP to interphase extracts, hGTSE1-GFP no longer tracks growing MT tips (Figure 3F, Movie S12), suggesting that CDK1 phosphorylation of GTSE1 may abolish +TIP activity. Under the same conditions, purified EB1 maintained robust tip tracking activity (Figure 3F, Movie S13). These results are consistent with cell-cycle dependent phosphorylation of GTSE1 confining +TIP activity to interphase, most likely through disruption of the EB1-GTSE1 interaction by phosphorylation around SxIP motifs. Interestingly, microtuble lattice-binding of GTSE1 may also be affected by cell cycle stage and CDK1 activity, as it was less prominent in mitotic extracts or after addition of cyclinBΔ90 to interphase extracts.

The interphase-specific tip-tracking activity of GTSE1 prompted us to examine potential effects of GTSE1 depletion on the interphase microtubule network and microtubule dynamics in human cells. First, we observed the microtubule network in fixed cells following RNAi depletion of GTSE1. Here we noticed that in a small fraction of GTSE1-depleted cells the microtubule network appeared slightly less radially organized, with microtubules appearing more dense, more curved and more randomly organized in relation to the cell center (Figure S2), although the degree and occurrence of this observation was inconsistent. To quantify potential defects in microtubule dynamics in interphase in live cells, as has been reported for several, but not all, +TIP proteins, we depleted GTSE1 by RNAi in human cells expressing a fluorescently tagged EB3 protein, which allows one to follow the dynamic properties of growing microtubules [46], [47]. By analyzing tracks of EB3 comets after GTSE1 RNAi, we found that in cells depleted of GTSE1, overall microtubule growth velocities were only slightly decreased (Figure S2). Additionally, the average track length of growing microtubules, as well as the number of tracks observed in GTSE1-depleted cells were only moderately decreased (Figure S2). Although these analyses showed that GTSE1 depletion does have a slight impact on microtubule polymerization dynamics, the minimal effects suggested that the primary microtubule-related role of GTSE1 in interphase cells may not be in regulating microtubule growth rates or the lifetime of growth events, but potentially in other parameters of microtubule function, or in tying dynamic microtubules to microtubule-dependent processes through the plus end.

### GTSE1 Modulates Cell Migration in an EB1-dependent Manner

To shed light on possible interphase roles for GTSE1 activity, we investigated the relative protein levels of GTSE1 in both non-transformed and transformed cell lines, and across the cell cycle. GTSE1 has been reported as significantly overexpressed in different tumors [48]–[50], suggesting that cell cycle misregulation and/or overexpression of an important GTSE1 activity may play a role in cancer progression. We initially monitored GTSE1 protein levels over a panel of non-transformed and transformed cell lines with different degrees of tumorigenicity (Figure S3A). Five different non-tumorigenic cell lines tested had very low GTSE1 levels. In contrast, all four tumorigenic cells lines tested had dramatically elevated GTSE1 protein levels, suggesting a potential correlation. GTSE1 expression has been shown in non-transformed cells to be most abundant during the S and G2 phases of the cell cycle [35]–[37]. To determine if the increased expression levels in the transformed cell lines was due to enrichment in any specific phase of the cell cycle relative to non-transformed cells, we FACS sorted Wi38 (non-transformed, human diploid fibroblast) and U2OS (transformed) cell lines into fractions enriched for cells in G1, S, or G2/M phases of the cell cycle. GTSE1 protein levels in each fraction were analyzed by western blot, normalized to actin levels. While this analysis showed that that GTSE1 levels were elevated across all cell cycle phases in the transformed cells, particularly noticeable was that in non-transformed cells, GTSE1 protein appears to be restricted to very low levels in G1, while in transformed cells, it is as abundant in G1 as it is in the maximal G2 levels of normal cells (Figure S3B, compare lanes 2, 4, and 6).

Invasion of cancer cells has been reported to occur preferentially in G1 phase of the cell cycle [51], and in several cell types studied the potential for migration is highest in G1 [52]–[54], suggesting that misregulation of proteins in G1 may stimulate migration. EB1 has been shown to be required for efficient migration in both nontransformed and transformed cells, and is expressed at similar levels throughout the cell cycle [23]–[25]. The increased G1 expression of GTSE1 in transformed cells, combined with the EB1-dependent localization of GTSE1 to growing microtubule ends, prompted us to ask whether GTSE1 levels may potentiate cell migration.

**Figure 4:**
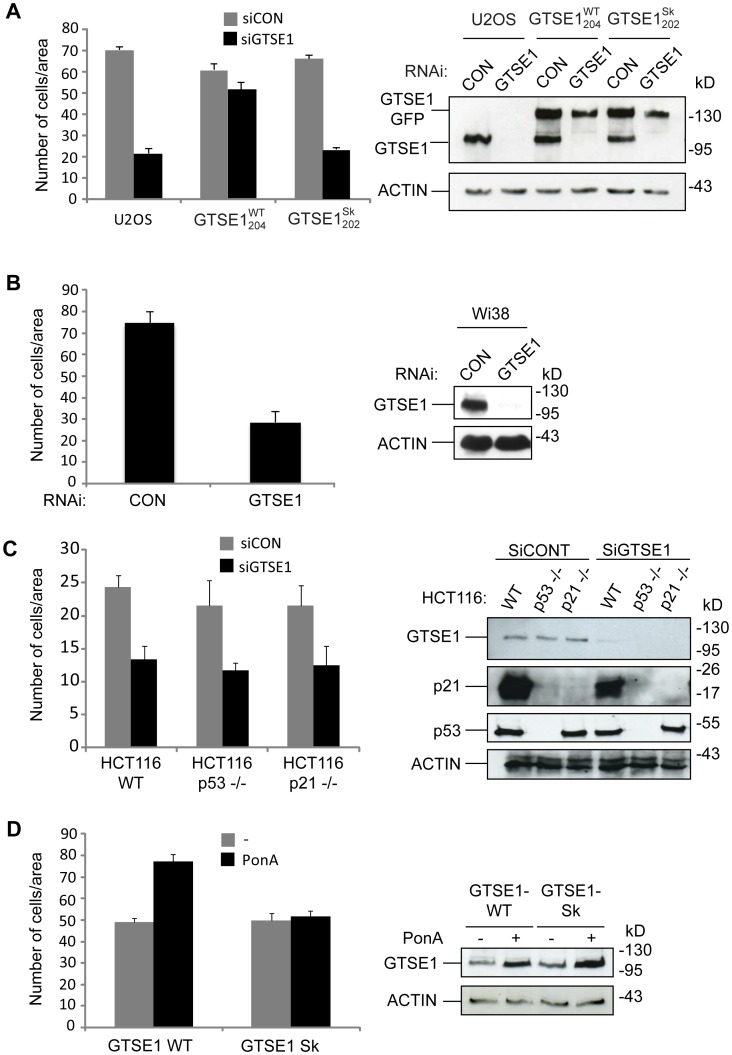
GTSE1 modulates cell migration in an EB1-dependent manner. (A) Transwell migration assay and western blot in U2OS cells, and U2OS cells stably expressing RNAi-resistant wild-type GTSE1-GFP (GTSE1^WT^_204_), or RNAi-resistant GTSE1-GFP mutated at the SKIP motifs (GTSE1^Sk^_202_). Cells were transfected with control (CON) or GTSE1 siRNA for 36 h and seeded on transwell membranes. Histograms show the mean number of cells/area that migrated through the transwell after 16 h (10 replicates/experiment). Error bars represent the standard error of the mean from three independent experiments. Western blots were performed on cells after the same treatment, and blotted with anti-GTSE1 and anti-actin. (B) Transwell migration assay and western blot in Wi38 cells. Cells were transfected and analysed as in (a). (C) Transwell migration assay and western blot in HCT116 wild type, HCT116 p53−/−, and HCT116 p21−/− cell lines. Cells were transfected and analyzed as in (a). Western blots were performed on cells after the same treatment, and blotted with anti-GTSE1, anti-p21, anti-p53, and anti-actin. (D) Transwell migration assay and western blot in H1299 cells containing inducible constructs for expression of wild type GTSE1 (GTSE1 WT) or SKIP-domain mutated GTSE1 (GTSE1 Sk). Cells were untreated (−) or treated with Ponasterone A (PonA) (+) for 24 h to induce GTSE1 expression, then trypsinized and seeded on transwell membranes. Histograms show the mean number of cells/area that migrated through the transwell after 16 h (10 replicates/experiment). Western blots were performed on cells after the same treatment, and blotted with anti-GTSE1 and anti-actin.

We first performed a wound-healing assay in U2OS cells in which cells are induced to migrate by creating a scratch in a confluent layer. While control-transfected cells almost completely invaded the wound after 36 hours, cells depleted of GTSE1 migrated into the wound more slowly (Figure S3C). As a more accurate means of measuring cell motility, we performed transwell-migration assays with the same cellular background. Again, depletion of GTSE1 by RNAi resulted in less migration than control cells (Figure 4A). Cell proliferation was not affected under these same conditions, indicating the observed effect was due to migration *per se* (Table S2). To determine if reducing GTSE1 levels would also affect migration in non-transformed cells, we depleted GTSE1 by RNAi in Wi38 cells, and measured transwell migration. Migration was significantly reduced in these cells as well (Figure 4B).

As GTSE1 regulates both p53 and p21 stability [55], we wished to exclude an indirect affect on cell migration through an altered p53 or p21 pathway in GTSE1-depleted cells. We therefore depleted GTSE1 by RNAi in HCT116 wt, HCT116 p53−/−, and HCT116 p21−/− cell lines. All three cell lines displayed a similarly reduced migratory capacity in transwell-migration assays (Figure 4C), suggesting a p53/p21 axis-independent effect.

If GTSE1 affected migration directly, we expected that overexpression of GTSE1 could potentially increase cells’ migratory capacity. To test this, we used an H1299 cell line inducible for GTSE1 expression (JPIC/H, [55]). Strikingly, after GTSE1 induction these cells acquired increased migratory capacity in transwell-migration assays, but did not increase proliferation (Figure 4D; Table S2), suggesting a direct role for GTSE1 in cell migration.

To test whether GTSE1’s ability to modulate cell migration was through its +TIP activity, we assayed its for the dependency on an interaction with EB1. First, an H1299 cell line inducible for GTSE1 was established as above, but with GTSE1 mutated at the SKIP domain (hGTSE1^Sk^). In contrast to the wildtype GTSE1, upon induction of the hGTSE1^Sk^ protein, cells did not increase their ability to migrate (Figure 4D). To confirm this result, we assayed migration in clonal U2OS BAC lines expressing siRNA-resistant transgenes encoding wild-type GTSE1 (GTSE1^WT^_204_) or GTSE1 mutated at the tandem EB1-binding SKIP-like motifs (GTSE1^Sk^_202_). GTSE1^WT^_204_ was able to largely recover the impairment in cell migration after depletion of the endogenous protein, confirming the specificity of the RNAi and the functionality of the GTSE1-GFP transgene (Figure 4A). In contract, GTSE1^Sk^_202_ did not rescue the defect, and after RNAi of the endogenous protein, migration was reduced to levels equivalent to a full depletion of GTSE1 (Figure 4A). Together, these results show that GTSE1 protein levels determine cell migratory capacity, and that the GTSE1-EB1 interaction (and hence +TIP activity) plays a fundamental role in GTSE1-dependent cell migration.

### GTSE1 is Required for Focal Adhesion Disassembly in an EB1-dependent Manner

**Figure 5:**
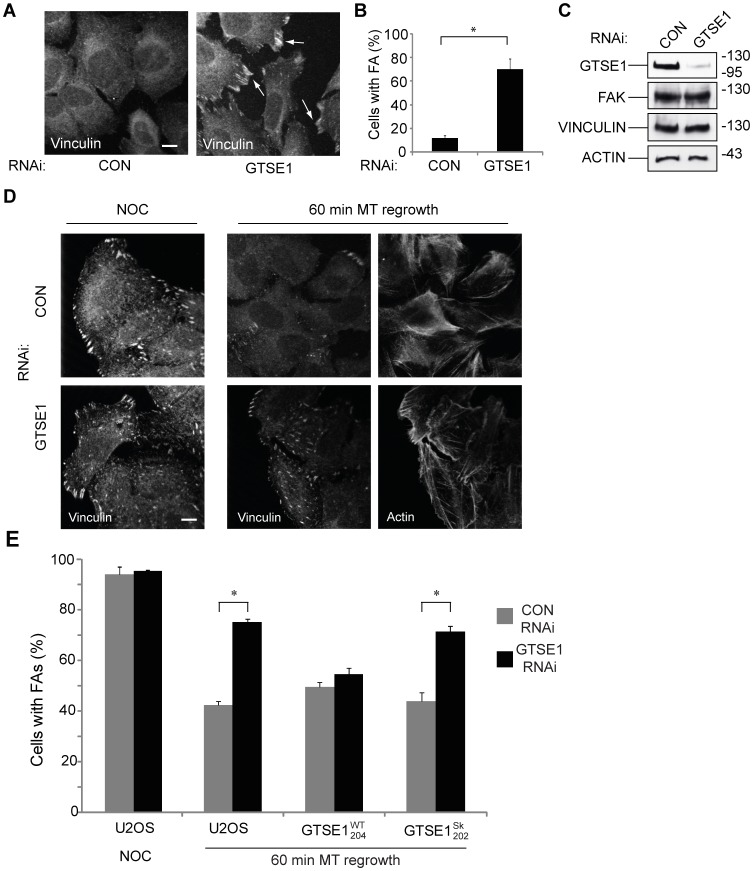
GTSE1 modulates focal adhesion disassembly in an EB1-dependent manner. (A) Immunofluorescence of U2OS cells transfected with control (CON) or GTSE1 siRNA for 24 h followed by serum starvation for 48 h, stained for vinculin. Focal adhesions persist in cells depleted of GTSE1. Scale bar represents 10 microns. (B) Quantification of focal adhesion (FA) disassembly from experiments from (A). The percentage of cells containing 10 or more focal adhesions after serum starvation-induced disassembly was determined (n = >50 cells per experiment, 3 experiments for each condition). * indicates p<0.05 as determined by a Student’s t test. (C) Western blot of U2OS cells transfected with control (CON) or GTSE1 siRNA and serum-starved for 48 h, blotted with anti-GTSE1, anti-FAK, anti-vinculin, and anti-actin. (D) Immunofluorescence of U2OS cells transfected with control (CON) or GTSE1 siRNA for 36 hours. Cells were imaged after treatment with nocodazole for 4 hours, and 60 minutes following washout of nocodazole to allow microtubule regrowth. Cells are stained for vinculin and actin. GTSE1-depleted cells contain more focal adhesions that wild-type following microtubule regrowth. Scale bar represents 10 microns. (E) Quantification of focal adhesion disassembly in U2OS cells, following the assay described in (D). Cells stably expressing RNAi-resistant wild-type GTSE1-GFP (GTSE1^WT^_204_) or GTSE1-GFP mutated at the SKIP motifs (GTSE1^Sk^_202_) were additionally assayed. Quantification was performed as described in (B). Cells containing only mutant GTSE1 unable to interact with EB1 or track growing microtubule ends are deficient for microtubule-dependent focal adhesion disassembly.

Focal adhesion disassembly is required for cell migration and dependent on microtubules. It is also significantly upregulated in G2 to prepare cells for rounding that takes place in mitosis. We have shown that GTSE1 is a MAP required for cell migration, and normally upregulated in G2 as well. To test if GTSE1 was required for focal adhesion disassembly, we depleted GTSE1 from serum-starved U2OS cells and counted focal adhesions. Serum-starved cells generally have relatively few focal adhesions (<20% of cells have more than 10 focal adhesions; Figure 5A,B). After GTSE1 depletion, we observed a dramatic increase in the number of FAs present as compared to control cells (Figure 5A,B). Under these conditions, GTSE1 protein was diminished by more than 80%, without affecting the levels of actin or the focal adhesion components FAK or vinculin (Figure 5C).

Because microtubules stimulate focal adhesion disassembly, treatment of cells with the microtubule polymerization-inhibiting drug nocodazole results in the persistence of focal adhesions, which are then disassembled when the drug is washed out and microtubule growth resumes [14]. We assayed for microtubule-dependent focal adhesion disassembly under these conditions. After nocodazole treatment, control transfected and GTSE1-depleted cells accumulated focal adhesions to a similar degree (Figure 5D, E). In contrast, following nocodazole washout and 60 minutes of microtubule regrowth, the number of focal adhesions in control transfected cells was reduced by about half, while in GTSE1-depleted cells there was only a small change (Figure 5D, E), indicating a defect in microtubule-dependent focal adhesion disassembly (Figure 5D, E). Consistent with the inhibition of focal adhesion disassembly, cells depleted of GTSE1 maintained stress fibers after microtubule regrowth, whereas cells treated with control siRNA had fewer stress fibers (Figure 5D). We did not detect changes in the degree of interphase microtubule regrowth from the centrosomes after cold and nocodazole treatment in cells depleted of GTSE1 (data not shown).

In addition to its previously defined role in p53 regulation and the DNA damage response, we have shown that GTSE1 localizes to the microtubule lattice and growing microtubule tips independently. To test whether the role of GTSE1 in focal adhesion turnover was dependent on its microtubule localization, and more specifically, on MT plus end localization and interaction with EB1, we performed the same assays for focal adhesion disassembly with cells containing GTSE1 mutated for interaction with EB1. The wild-type RNAi-resistant GTSE1-GFP construct (GTSE1^WT^_204_) was able to rescue the defect in focal adhesion disassembly caused by depletion of GTSE1, but in the SKIP domain-mutated construct (GTSE1^Sk^_202_) there was no distinguishable difference in focal adhesion numbers from GTSE1-depleted U2OS cells (Figure 5E). Together, these data indicate that GTSE1 is required for focal adhesion disassembly induced by MTs, and that the GTSE1–EB1 interaction is critical in this process.

### GTSE1 Expression in Breast Cancers Correlates with Time to Metastasis, Invasiveness and Clinical Outcome

We have shown that GTSE1 is overexpressed in several transformed cell lines with respect to non-transformed cell lines (Figure S3). To identify any potential clinical cancer-related correlations with GTSE1 expression, we screened for associations with any tumor–relevant conditions using the Oncomine cancer microarray database (www.oncomine.org) [56] (Figure S4A). This screening identified 61 unique analyses over all cancers where GTSE1 expression showed significantly higher expression in tumor tissues as compared to normal tissues. Notably, clinical outcome correlated with increased GTSE1 expression in 12 cases, 10 of which were in breast cancer.

**Figure 6:**
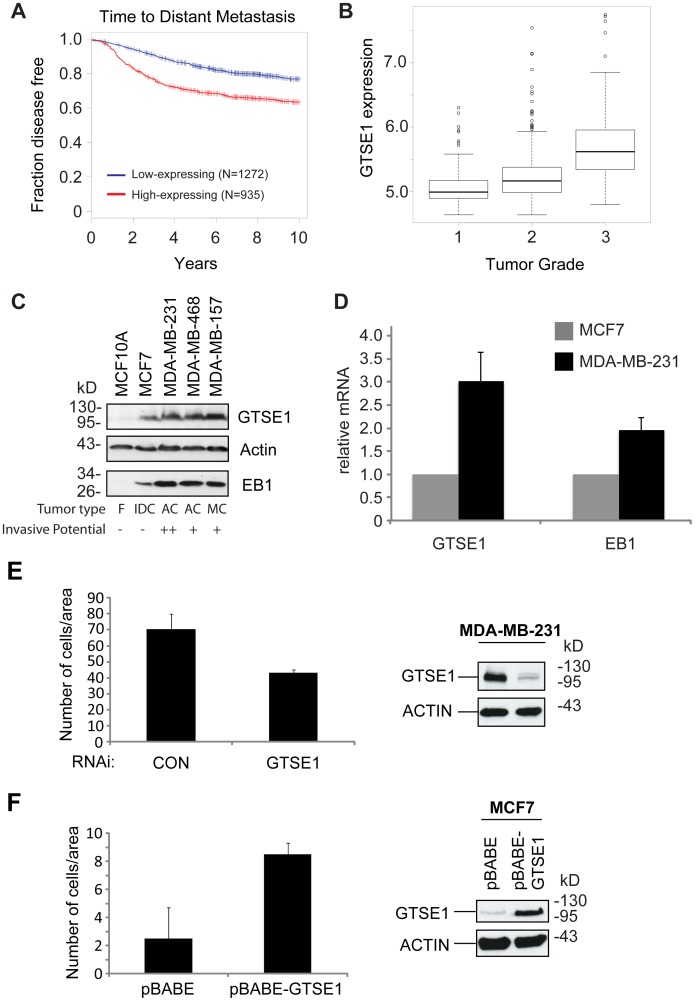
GTSE1 expression in breast cancer tumors and cells correlates with time to metastasis and invasiveness. (A) Kaplan–Meier survival curve of time to distant metastasis of breast cancer patients classified according to the expression of GTSE1. Red line: cases with high expression of GTSE1, blue line: cases with low expression of GTSE1. (p-value <10?–15) (B) Boxplots of the distribution of gene expression intensities of GTSE1 across different breast cancer subtypes (Grade 1, 2 or 3; p<10?-5; linear regression analysis),. (C) Western blot analysis of GTSE1 and EB1 protein levels in different breast cancer cell lines. Tumor types are: F, fibrocystic disease, non-transformed, immortal cell line; IDC, invasive ductal carcinoma; AC, adenocarcinoma; MC, metaplastic carcinoma. Invasive potential is characterized as not invasive (−), invasive (+), or highly invasive (++). Adapted from Neve et al. Cancer Cell 2006. (D) Quantitative RT-PCR analysis of GTSE1 and EB1 relative mRNA levels in MCF7 and MDA-MB-231 cells. Error bars represent the standard error of the mean from three independent experiments. p<0.01 (Student’s t-test). (E) Transwell migration assay and western blot of the MDA-MB-231 cell line. Cells were transfected with control (CON) or GTSE1 siRNA for 36 hours, trypsinized, and seeded on transwell membranes. Histograms show the mean number of cells/area that migrated through the transwell after 16 h (10 replicates/experiment). Error bars represent the standard error of the mean from three independent experiments. * indicates p<0.05 (Student’s t-test). Western blots were performed on cells after the same treatment, and blotted with anti-GTSE1 and anti-actin. (F) Transwell migration assay and western blot of the MCF7 cell line containing a stably integrated GTSE1 overexpression construct (pBABE-GTSE1) or empty vector (pBABE). Cells were trypsinized and seeded on transwell membranes. Histograms show the mean number of cells/area that migrated through the transwell after 16 h (10 replicates/experiment). Western blots were performed on cells after the same treatment, and blotted with anti-GTSE1 and anti-actin.

To delve deeper into clinical correlations with GTSE1 expression, we analyzed several microarray data sets of breast cancer, collectively consisting of more than 2000 patients. Kaplan-Meier survival analysis of the combined data sets showed that breast cancer patients expressing higher GTSE1 levels in tumors displayed both shorter survival time (p<10^−9^; Fig. S4B) and a shorter time to distant metastasis (p<10^−15^; Fig. 6A, Figure S4C). We also found a significant correlation between GTSE1 expression and the grade of breast cancers, with the most invasive and aggressive cancers (Grade 3) showing highest expression of GTSE1 (Figure 6B). Together these data show a correlative relationship between the misregulation and overexpression of GTSE1 found in tumors, and tumor invasiveness and prognosis.

To investigate the significance of GTSE1 expression levels in the context of breast cancer cell lines, we next analyzed a panel of lines of varying breast cancer tumor types with different degrees of invasive potential for both GTSE1 and EB1 protein levels. As is shown in Figure 6C, two lines with low invasive potential, MCF10A and MCF7, have relatively low GTSE1 and EB1 protein levels compared to three highly invasive lines, MDA-MB-231, MDA-MB-468, and MDA-MB-157.

We next looked for a causal link between GTSE1 expression and migratory ability in two breast cancer cell lines. The MCF-7 cell line is a non-tumorigenic, non-invasive breast cancer line with very low invasiveness and migratory capability, often used to study potential factors that stimulate metastasis [57]. In contrast, MBA-MD-231 is a highly metastatic, invasive and tumorigenic breast cancer cell line with a relatively high migratory capacity. In addition to containing higher protein levels for both GTSE1 and EB1 (Figure 6C), the more invasive MBA-MD-231 cells had 3-fold more GTSE1 mRNA expression as MCF7 cells as measured by RT-PCR, with EB1 also significantly increased. Transwell migration assays on these cell lines confirmed their expected migratory abilities (Figure 6E, F). We next tested whether modulating GTSE1 expression levels in these lines would correspondingly affect their migratory ability. Indeed, siRNA-mediated reduction in MBA-MD-231 cells significantly reduced their migration (Figure 6E). Furthermore, increasing GTSE1 expression levels through a retroviral vector in the poorly invasive MCF-7 cell line dramatically increased its migration to levels 4-fold higher than control cells (Figure 6F). In both cases, cell proliferation was not affected (Table S2). Thus GTSE1 protein levels correlate with invasiveness and metastasis in clinical breast cancer tumors, and determine cell migratory capacity in breast cancer cell lines.

## Discussion

Here we have identified GTSE1 as a microtubule-associated and plus-end tracking protein required to promote cell migration. GTSE1 localizes to growing microtubule tips through interaction with the EB1+TIP, and this interaction and localization is required for GTSE1’s role in cell migration, as well as for turnover of focal adhesion complexes.

Depletion of EB1 from cells has been shown to affect cell migration [23]–[25], but this effect has been mostly ascribed to MT interaction and stabilization at the cell cortex at the leading edge of migrating cells. Here we have identified the EB1-dependent +TIP activity of GTSE1, which is required for its role in cell migration, as also required to support microtubule-dependent disassembly of focal adhesions. Several elegant studies have demonstrated that microtubule targeting of focal adhesions induces their disassembly [12]–[14], [58]. One +TIP, ACF7, has been previously shown to play a role in focal adhesion disassembly. ACF7 binds both actin and microtubules and is proposed to mediate the targeting of MTs to focal adhesions by guiding them along F-actin [28], [31]. However, the mechanism by which focal adhesions are then triggered to disassemble remains elusive. Clathrin-mediated endocytosis has emerged as an important step in this process [58], [59]. One hypothesis is that microtubules serve as tracks to deliver undefined “relaxing factors” via motor proteins, which eventually lead to clathrin endocytosis of focal adhesion complexes [12], [60]. Alternatively, or in combination, proteins located at the growing plus tip would be ideally positioned to directly activate focal adhesion disassembly. In this context, GTSE1’s requirement for focal adhesion disassembly is intriguing, considering that it is not only a +TIP, but also clearly associates with clathrin complexes. We have previously shown by affinity-purification mass spectrometry analysis that GTSE1 significantly associates with a large number of clathrin subunits and clathrin-associated proteins, both in mitosis [38] and interphase (unpublished data). GTSE1 is also enriched in clathrin heavy chain immunoprecipitations in mitosis [38] and found associated with clathrin cages in interphase [39]. It will therefore be interesting in future studies to precisely define a role of the GTSE1-clathrin interaction in FA disassembly.

It is also possible that the effect of GTSE1 on focal adhesion disassembly could be through modulation of microtubule dynamics, which then affects the targeting dynamics. Overexpression of GTSE1 has a clear effect of increasing cell migratory capacity, indicating the mechanism by which it acts supports gain of function alteration. After GTSE1 depletion, we did observe a small reduction in microtubule growth velocity, as well as the number of growth events and length of growth events. The degree to which these properties were reduced (∼10–15%) were, however, moderate when compared to the reduction after depletion of other +TIPs known to inhibit microtubule dynamics, such as SLAIN2 and Ch-Tog (60% and 40% reduction in growth velocity, respectively) [21], [47]. Nevertheless, we cannot rule out that these changes may contribute to the focal adhesion disassembly defect, or that other aspects of microtubule dynamics or stability are affected. We did observe subtle changes in the overall organization of the MT lattice after depletion of GTSE1, similar to those reported after depletion of SLAIN2 or ch-TOG [21]. SLAIN2 interacts with ch-TOG and modulates its localization to growing microtubule plus ends. Ch-TOG also co-immunoprecipitates with GTSE1 [38], suggesting a potential overlap in the function of these proteins.

Consistent with a specialized role for its +TIP activity, GTSE1 does not track growing microtubule tips during mitosis, when cells are rounded up and immotile, even though it is at peak expression levels. The confinement of GTSE1+TIP activity to interphase is likely the result of the mitotic phosphorylation events at residues around the EB1 interaction domain, which could disrupt the interaction between GTSE1 and EB1, such that it is no longer recruited to growing microtubule tips. Similar regulation of plus-end tracking and EB1 interaction has been reported for other +TIPs [18], [20]–[22]. Our studies point to the cell cycle kinase CDK1 as playing a role in regulating GTSE1 plus-end tracking, but we cannot rule out an indirect effect. In human cells, the timing of the loss and recurrence of GTSE1+TIP activity correlates well with CDK1 activity, particularly evident in the rapid onset of tip tracking at anaphase onset (Movie S9). The phosphorylation of the +TIP SLAIN2 that disrupts the interaction with EB1 during mitosis contains the same conserved CDK1 sites (Figure 2A), and was also shown to be CDK1 dependent, indicating a common mechanism [21].

As normal cells transition from mitosis into G1, the high levels of GTSE1 protein are returned to very low levels by degradation through Cdh1-APC [35], [36], [61]. GTSE1 only starts to become abundant again in S phase, and protein levels increase through G2 and into prophase, where focal adhesion disassembly and cell rounding is at a peak. Thus, GTSE1+TIP activity appears to be carefully regulated by the cell to reach maximum levels in the S and G2 phases in two ways: through inhibitory phosphorylation in mitosis and through cell-cycle dependent expression control.

In this regard, it is notable that sequence analysis of GTSE1 shows that large portions of GTSE1 are likely intrinsically disordered. Intrinsically disordered proteins (IDPs) are often involved in signaling and regulatory functions, and many have been implicated as misregulated in human diseases [62], [63]. This relates to the observation that IDPs are often subject to tight regulation in cells, through control of protein synthesis, degradation, and post-translational modification. Due to the nature of their disordered sequences, IDPs are particularly sensitive to misregulation. Inappropriate protein levels and activities of IDPs at times and places in the cell outside of their normally controlled functions often result in the alteration of protein interactions and signaling pathways. The inappropriately high levels of GTSE1 expression in G1 in transformed cells indicates loss of its normally tight regulatory pathways, as occurs with many IDPs. Similarly, the very tightly controlled low expression levels of GTSE1 in G1 in normal cells may suggest that at this stage cells are particularly sensitive to modulators of focal adhesion disassembly affecting migration. It can thus be hypothesized that transformed cells are hijacking a primarily G2-restricted function of increased focal adhesion dynamics in normal cells, by maintaining increased GTSE1 levels in G1 to sustain high levels of unregulated migration and invasion.

Interestingly, upregulation of GTSE1 expression was identified as a potential marker for metastasis in oral tongue squamous cell carcinoma [48]. More recently, GTSE1 was identified as one of three cell cycle regulatory genes (along with CDKN3 and Cyclin B1) whose upregulation in gastroenteropancreatic neuroendocrine tumors correlate with metastasis [50]. These observations are consistent with our finding that in breast cancer patients, GTSE1 mRNA expression levels correlate with time to metastasis and tumor grade. Two major hallmarks of cells that have acquired metastatic capabilities are loss of adhesion properties and an increase in cell motility, which together help to promote invasion as well as angiogenesis [1]–[4]. Here we have shown that the molecular activity of GTSE1 leading to stimulation of cell migration and loss of focal adhesions is EB1-dependent microtubule plus-end tracking, providing an intriguing link between microtubule plus-end functions and metastasis.

## Materials and Methods

### Cloning and Plasmids

The BAC RP11-1152E11 containing hGTSE1 was purchased from BACPAC Resources Center. A ‘LAP’ tag cassette [64] was recombined at the C-terminus of hGTSE1 by Red E/T-based recombination [65]. Point mutations were introduced in the hGTSE1 BAC through counterselection recombineering based on an RpsL-amp cassette and dual Redβ and Redγ expression (pABRG) as described [40]. The RNAi-resistant GTSE1 mutation (contained in GTSE1^WT^_204_ and GTSE1^Sk^_202_) changed the siRNA target site GATTCATACAGGAGUCAAA to GGTTTATCCAAGAAAGTAA.

pcDNA3-HA-hGTSE1 was previously described [33]. GST-hGTSE1 contains the full-length hGTSE1 fused to GST (pGEX-4T1, GE Healthcare). The construct pIND-hGTSE1 used to generate PonA - inducible cell lines was previously described [55]. pBABE-Puro-hGTSE1 was constructed by subcloning full-length hGTSE1 into the pBABE-Puro retroviral vector. pEGFP N1-EB1 and pGEX (6p-2)-EB1 [66] were a generous gift from Bert Vogelstein. pEGFP N1-EB1-N and pEGFP N1-EB1-C encode for deletion mutants of EB1 from amino acids 1–143 and 143–268 respectively, and were generated by PCR using pEGFP N1-EB1 as the template.

### Cell Lines and Cell Culture

Wi38, MCF10A, U2OS and H1299 cell lines were obtained from ATCC. All cell lines were grown in DMEM containing 10% fetal bovine serum, 2 mM L-glutamine, 100 U/ml penicillin and 100 µg/ml streptomycin at 37°C and 5% CO2, with the exception of the H1299 (JPIC/H) cell line (RPMI-1640 medium), R1/E (DMEM, 20% FCS, 50 µM beta-Mercaptoethanol, 1x non-essential amino acids (Invitrogen), 100 U/ml penicillin, 100 mg/ml streptomycin, and 13 ng/ml mouse LIF (Leukemia inhibitory factor)), Wi38 (MEM 10%FBS and non essential aminoacids), MCF10A (DMEM/F12 5% horse serum, 10 mM HEPES, 20 ng/ml EGF, 100 ng/ml cholera toxin, 0,01 mg/ml insulin and 500 ng/ml hydrocortisone) and MASC [67] (60% DMEM 1 mg/ml glucose, 40% MCDB-201, 2% FBS, 1 mg/ml linoleic acid, 10 nM dexamethasone, 0,1 mM ascorbic acid-2-phosphate, 0,01 mg/ml insulin, 10 ng/ul PDGF and 10 ng/ul EGF). HCT116 p53−/−, HCT116 p21−/− and parental cells [68] were a generous gift from Bert Vogelstein. To induce hGTSE1 expression in JPIC/H cells [55], Ponasterone A, a synthetic analog of ecdysone (Invitrogen) was added to the culture medium at a final concentration of 5 µM for 24 h.

BAC constructs, mCherry-alpha-tubulin (puro) plasmid, and EB3-mCherry (puro) plasmid were transfected into U2OS cells in 6 cm dishes with 20 ul Effectene (Qiagen) following the manufacturer’s protocol, stable line populations selected on G418 or puromycin, and individual clones isolated. The calcium phosphate method was used to transfect 293 cells with constructs for immunoprecipitation experiments. FuGENE 6 (Roche Diagnostics) was used to transfect cells with pIND vectors to generate inducible cell lines.

Cell sorting of U2OS and Wi38 cells into G1, S and G2/M fractions was performed by tripsinizing and fixing cells in 70% ethanol. Cells were subsequently washed and stained with TO-PRO3 iodide (Invitrogen) and sorted on a FACSAria III (BD Biosciences).

### RNAi interference

siRNAs against hGTSE1 used were GAUUCAUACAGGAGUCAA (sequence used to design RNAi-resistant BAC constructs; from Applied Biosystems) and AAAUUUGACUUCGAUCUUUCA (MWG Biotec). siRNA against EB1 was UUCGUUCAGUGGUUCAAGA (Applied Biosystems). Control (“CON” ) siRNAs used were either Silencer Negative Control #3 (Applied Biosystems) or LacZ-targeting (GUGACCAGCGAAUACCUGU; MWG Biotec). siRNAs were transfected using Oligofectamine (Invitrogen), Lipofectamine RNAi MAX (Invitrogen), or X-tremeGENE siRNA Transfection Reagent (Roche Diagnostics) as recommended by the manufacturer at a final concentration of 80–120 nM. For all transfections media was changed after 6–8 hours. Cells transfected with siRNA were analyzed after 36 h unless stated otherwise.

### Antibodies

Rabbit antibodies against human hGTSE1 were either as previously described [37], or generated in rabbits using a purified GST-fusion protein of amino acids 166–257. For affinity purification, the same regions were cloned in frame to MPB in the pMAL-c2 vector and the fusion proteins purified essentially as suggested by the manufacturer (New England Biolabs). The MBP-fusion proteins were coupled to 1 ml NHS HiTrap columns (Amersham Pharmacia Biotech) and affinity purification performed using standard procedure.

The following antibodies were obtained from commercial sources: rabbit Anti-FAK (Cell Signaling), mouse Anti- Vinculin (VIN-11-5, Sigma), mouse anti-EB1 (BD Biosciences), mouse anti-Alpha-tubulin (DM1a, Sigma), mouse anti-HA 12CA5 (Roche), rabbit anti-Actin (Sigma), rabbit anti-p21 (C-19, Santa Cruz Biotechnology), mouse anti-p53 (DO-1, Santa Cruz Biotechnology).

### Protein Purification

Full length human GTSE1 was tagged at the c-terminus with a tandem GFP-his tag. This construct expressed in SF+ cells using the Bac-to-Bac system from Invitrogen. Virus was prepared according to the provided protocol, except baculovirus infected insect cell (BIIC) stocks were made as described [69]. 200 µl of BIIC stock was used to infect 500 mL of SF+ cell culture at 1×10^∧^6 cells/mL. Cells were harvested 72 hours after infection by centrifugation at 1700 rpm for 15 min in a Hereaus Megafuge centrifuge. Cells were resuspended in ice-cold lysis buffer (50 mM HEPES pH 7.5, 200 mM NaCl, 5% glycerol, 0.1% Triton-X-100) and snap frozen in liquid nitrogen. After the cells were thawed, a complete protease inhibitor cocktail tablet (Roche), 3.6 µg/ml E64 protease inhibitor, 1 µg/ml PMSF, 1 µg/ml pepstatin A, and 10 mM CaCl_2_ were added. The resuspended cells were homogenized using a Dounce homogenizer. The crude lysate was clarified by centrifugation at 80,000 rpm for 45 min in a Beckman Ultra-max centrifuge with a MLA-80 rotor and loaded onto a Ni2–sepharose HisTrap HP column (GE Healthcare), equilibrated with imidazole buffer (50 mM TrisHCl buffer pH 8.0, 300 mM NaCl, 15 mM imidazole, 10% glycerol). The column was washed with 30 mM imidazole and 60 mM imidazole buffers and GTSE1-GFP-his was eluted with 600 mM imidazole. Peak Ni-column fractions were pooled and passed through a size exclusion chromatography column (GE Healthcare Superdex 200 16/60) pre-equilibrated with elution buffer: 10 mM TrisHCl, 10 mM Bis-tris, 100 mM KCl, pH 6.6. Peak fractions were pooled and concentrated to at least 6 µM using an Amicon Ultra 10 K MWCO concentrator (Millipore). Protein was stored with glycerol added to 10% and DTT added to 1 mM. Tubulin was purified from porcine brain as described [70].

### Cell Migration Assays

For wound-closure experiments, U2OS cells were plated in 6-well plates and cultured to confluence. Cells were scraped with a pipette tip, washed with PBS to remove debris, and 0.1% serum medium was added to allow wound healing. Phase-contrast images of the wound were taken immediately after wounding and at the same location after 36 hours.

Transwell assays were performed in 24 well 8 µm PET inserts (BD Falcon). Briefly, 1×10^5^ cells were seeded on the top of the transwell membrane in serum-free medium, and the lower compartment was filled with 10% serum medium. Cells were allowed to migrate for 16 hours. Cells in the upper part of the transwells were removed with a cotton swab; migrated cells were fixed in PFA 3% and stained with Crystal Violet 0.5%. Filters were photographed and migrated cells were counted in 10 randomized fields. (Every experiment was repeated at least three times independently.).

### Immunofluorescence

Cells were seeded on glass coverslips in 3 cm culture dishes. After washing with PBS, cells were fixed in 3% paraformaldehyde in PBS, treated with 1% glycine in PBS, and permeabilized in 0.1% Triton X-100 in PBS. The staining was performed using specific antibodies incubated in 5% bovine serum albumin in PBS at 37°C followed by fluorescein isothiocyanate or tetramethylrhodamine isothiocyanate-conjugated secondary antibodies (Sigma). DNA was stained with Hoechst (Sigma), and actin with fluorescein isothiocyanate-coniugated phalloidin (Sigma).

### Microscopy

Fixed cell fluorescence images were acquired on a Zeiss LSM 510 Meta confocal microscope with a 100×1.4 NA objective. For live cell imaging, cells were incubated in CO2-independent medium (Gibco) at 37 degrees. Live images were acquired with a CCD camera (CoolSNAP HQ, Roper Scientific) using a Deltavision RT imaging system (Applied Precision) (Olympus IX71) with a 100×1.35 NA UPLanApo or 60×1.42 NA PlanApo N objective. For live DNA visualization, Hoechst 33342(100 ng/ml) was added to the media one hour before imaging.

The total-internal-reflection fluorescence (TIRF) imaging was performed with a setup described previously [71]. The setup incorporates an Andor DV887 iXon camera on a Zeiss Axiovert 200 M microscope using a Zeiss 100X/1.45 a Plan-FLUAR objective. Standard filter sets were used to visualize tetramethylrhodamine, Alexafluor 488, and GFP.

### Immunoprecipitation and Western Blot Analysis

Cells were harvested in ice-cold lysis buffer containing 50 mM Tris-HCl, pH 8, 150 mM NaCl, 1% Nonidet P-40, 0.1 mM sodium orthovanadate, 2 mM dithiothreitol, 0.1 mM phenylmethylsulfonyl fluoride, 5 mM EDTA and Protease Inhibitor Cocktail (Sigma). After 10 min of rocking at 4°C lysates were clarified by centrifugation and precleared with 25 µl of Protein A-Sepharose CL-4B or GammaBind G Sepharose (Amersham Biosciences). Then, antibody prebound to 25 µl of Protein A-Sepharose CL-4B (for anti-hGTSE1 and anti-HA immunoprecipitations) or GammaBind G Sepharose (for anti-GFP immunoprecipitations) was added and incubated at 4°C for 2 h. The resin was washed and bound proteins were eluted in SDS-PAGE sample buffer. Western blot analysis was performed according to the standard procedures. Bound primary antibodies were visualized by enhanced chemiluminescence (ECL; Amersham Biosciences) after addition of horseradish peroxidase-conjugated secondary antibodies.

### Pull-down Binding Assays

#### *In vitro*

^35^S-labeled proteins were *in vitro* translated using TNT Quick Coupled Transcription/Translation System (*in vitro* protein expression) (Promega) and incubated with purified GST, GST-hGTSE1 or GST-EB1 (immobilized on glutathione-Sepharose 4B beads, Amersham Biosciences) in pull-down buffer (150 mM NaCl, 20 mM Hepes pH 7.5, 0,05% NP-40, 10% Glycerol, 0.1 mM phenylmethylsulfonyl fluoride and Protease Inhibitor Cocktail). Bound proteins were eluted and resolved on SDS-PAGE.

#### *In vivo*

Cells were lysed in buffer A (50 mM Tris-HCl pH 7.5, 300 mM NaCl, 0.5% NP-40, 10% glycerol, 0.1 mM phenylmethylsulfonyl fluoride and Protease Inhibitor Cocktail). Samples were clarified by centrifugation and an equal volume of buffer B (50 mM Tris-HCl pH 7.5, 0.5% NP-40, 10% glycerol, 0.1 mM phenylmethylsulfonyl fluoride and Protease Inhibitor Cocktail) was added. Lysates were incubated with purified GST or GST-hGTSE1 immobilized on glutathione-Sepharose 4B beads. Bound proteins were eluted and resolved on SDS-PAGE.

### Microtubule Co-sedimentation Assay

Plasmid DNA was translated *in vitro* as described in “Pull-down binding assay”. 10 µl of ^35^S-labeled *in vitro* translated protein were incubated with 50 µg of Taxol-stabilized microtubules [reconstituted according to the manufacturer’s instructions (Cytoskeleton Inc)], or in Taxol-containing microtubule buffer, in a total volume of 100 µl, for 30 min at 37°C. Samples were then centrifuged for 30 min at 100,000 g in an Airfuge and supernatants and pellets were resolved on SDS-PAGE and analyzed by autoradiography.

### Imaging Purified GTSE1 on Microtubules by TIRF Microscopy

Reaction channels were first rinsed with BRB80∶80 mM PIPES at pH 6.9, 1 mM MgCl2, and 1 mM EGTA. Reaction channels were incubated with 1% anti-rhodamine antibody (Invitrogen) in BRB80 for 5 min, followed by 1% pluronic F127 (Sigma) in BRB80 for 5 min, and finally rhodamine-labeled, GMPCPP stabilized microtubule seeds for 15 min. Channels were washed once with BRB80 and once with imaging buffer: BRB80 supplemented with 75 mM KCl, 0.1 mg/ml BSA, 1% b-mercaptoethanol, 40 mM glucose, 40 mg/ml glucose oxidase, and 16 mg/ml catalase. The microtubule seeds were placed under the TIRF microscope for viewing. An objective heater was used (Zeiss) to warm the sample to 35°C. 200 nM GTSE1-GFP was then added to the imaging buffer as well as 10 uM Tubulin and 1 mM GTP. Images were acquired at 5 second intervals.

### Imaging Microtubule Dynamics in *Xenopus* Egg Extracts by TIRF Microscopy

The total-internal-reflection fluorescence imaging setup and the preparation of chambers was performed as described above. CSF-extract from *Xenopus* eggs was prepared as described in [72]. Interphase was induced in extracts by adding 0.6 mM CaCl_2_ and cycloheximide (final conc. 100 µg/ml). CyclinBΔ90 was expressed and purified as in [73]. Before imaging, channels were washed once with BRB80, once with CSF-XB (10 mM HEPES, pH 7.7, 2 mM MgCl_2_, 0.1 mM CaCl_2_, 100 mM KCl, 5 mM EGTA, 50 mM sucrose), and imaged at 20°C. The extract was supplemented with CytochalasinD (final conc. 10 µg/ml) (addition of oxygen scavengers was not necessary). Due to microtubule severing activities in extracts, the GMPCPP seeds quickly become unstable. Therefore, handling and imaging must be within 3 minutes of adding extract.

### Focal Adhesion Assays

U2OS cell were treated with control (CON) or GTSE1 RNAi for 24 hours. For assays after serum starvation, cells were starved for an additional 48 h. For assays after microtubule regrowth, cells were treated with 10 µM nocodazole for 4 h to completely depolymerize MTs. Nocodazole was then washed out with serum-free medium, and MTs were allowed to repolymerize for 1 h. For both assays, cells were stained with anti-vinculin antibodies by immunofluorescence to visualize focal adhesions. To quantify focal adhesion disassembly, cells were scored positive if they retained 10 or more focal adhesions. Data are from three independent experiments in which more than fifty cells were analyzed for each condition in each experiment.

### Quantitative RT-PCR

Total RNA was extracted with QIAzol Lysis Reagent (Qiagen) and cDNA was transcribed with a QuantiTect Reverse Transcription Kit (Qiagen), according to the manufacturer’s instructions. GTSE1 and EB1 mRNAs were amplified with gene-specific primers and normalized to the B-actin mRNA level. Real-time PCR was performed with SYBR Green PCR Master Mix (Applied Biosystems) and StepOnePlus real time PCR machine (Applied Biosystems). Oligonucleotides used were GTSE FW - GCCCCGGGTGCTGTCAATGT; GTSE Rev - GCCCACTGCTGGGGATGTGC; EB1 FW – ACCCTGGTGTGGGCAACGGA; EB1 Rev – TCCCCCTCGTTCTCCTGGCAA; B-act FW – CCAACCGCGAGAAGATGA; B-act Rev – CCAGAGGCGTACAGGGATAG.

### Analysis of GTSE1 Expression in Cancers

For Oncomine analyses, GTSE1 expression was analyzed through the Oncomine Pro web tool using suggested standard parameters. Custom concept analysis was performed, and the “Summary view” (adapted) was reported.

For survival analysis from breast cancer data, several published gene expression datasets were considered and compared. The raw data were retrieved from the gene expression omnibus (GEO) public gene expression database (GSE1456, GSE4922, GSE5327, GSE6532, GSE7390, GSE11121, GSE12093, GSE2603, GSE16446, GSE19615, GSE20685, GSE21653). Data were normalized in R/Bioconductor environment using the RMA normalization method (affy package), creating a breast cancer meta-dataset. Gene annotation was obtained from brainarray custom CDF metadata packages, and the probesets were converted to Entrez Gene Id and Symbol Id. Each dataset was analyzed separately to avoid platform and signal merging problems, and only the results were combined together. To verify the correlation of the GTSE1 expression and breast cancer clinical data, a Mantel-Haenszel test was applied to the normalized meta-dataset (survival R package), and the Kaplan–Meier survival curve of time to distant metastasis (TDM) of breast cancer patients classified according to the expression of GTSE1 signature was obtained. With the same meta-dataset, we searched for the distribution of the gene expression intensities of GTSE1 signature across different breast cancer subtypes (stats R package).

### Mass Spectrometry

HeLa cells were arrested into mitosis using a double thymidine block followed by nocodazole treatment. Cells were harvested, lysed and proteins were digested with trypsin using the FASP procedure [74]. Phosphorylated peptides were enriched using a combination of strong cation exchange and TiO2 enrichment. Subsequently, enriched peptides were separated by nano-reversed phase liquid chromatography and eluting peptides were analyzed injected directly into an Orbitrap Velos mass spectromter (Thermo Scientific). Peptides were analyzed using TOP10 peptide sequencing with HCD fragmentation. Resolution of full-scan MS spectra was set to 60,000, of MS/MS spectra to 7,500. Raw data was analyzed using the open source MaxQuant software suit against the human IPI database (v.3.68). Phosphorylations of serine, threonine and tyrosin as well as methionine oxidations and N-terminal acetylations were set as variable, cysteine carbaminomethylations as fixed modifications. Peptide, protein and site-FDRs were set to 0.01. Only class I phosphosites with a localization probability higher than 75% were considered as identified.

### EB3 Tracking Analysis

Time-lapse imaging of the plus-end marker EB3-mEGFP stably expressed in HeLa cells was performed with the Zen2010 software on a LSM780 confocal microscope with a 63×PlanApochromat oil-objective NA 1.4 (Carl Zeiss) with a time resolution of 700 ms. A MATLAB program tracked the tips of the growing microtubules in interphase cells and concatenated them to microtubule tracks and derived track parameters such as microtubule track length, track number, and velocity. Details of tracking algorithm have been described previously [47]. Box plots were produced using MATLAB. Time projection was generated with ImageJ.

## Supporting Information

Figure S1 **Analysis of GTSE1 interaction with EB1.** (A) HEK293T cells were transfected with GFP-tagged EB1 and HA-tagged hGTSE1 for 24 h followed by immunoprecipitation using an anti-HA antibody. Western blots were performed by using anti-HA and -GFP antibodies. * indicates immunoglobulin heavy chain. (B) HEK293T cells were transfected with vectors encoding EB1 deletion mutants (EB1, EB1 N, EB1 C) and HA-hGTSE1, followed by immunoprecipitation using an anti-HA antibody. Anti-GFP and anti-HA antibodies were used for the immunoblot. (C) In vitro pull-down binding assay using recombinant/purified GST and GST-GTSE1 fusion proteins incubated with in vitro translated 35S-labeled EB1 (EB1 IVT). IVT proteins were visualized by autoradiography (Input shows 20% of the EB1 IVT input). Recombinant protein loading was checked by Comassie staining. (D) In vitro pull-down binding assay using recombinant/purified GST and GST-EB1 fusion proteins incubated with in vitro translated 35S-labeled human GTSE1 (hGTSE1), murine GTSE1 (mGTSE1) or Xenopus GTSE1 (xGTSE1).
(TIF)

Figure S2 **Analysis of MT dynamics after GTSE1 RNAi.** (A) Immunofluorescence of U2OS cells transfected with control (CON) or GTSE1 siRNA and stained for alpha-tubulin. (B-E) HeLa cells expressing the plus-end marker EB3-mEGFP were transfected with control or GTSE1 siRNA, and time-lapse imaging performed on interphase cells. (B) Box plot showing EB3 track velocities. (C) Box plot showing number of EB3 tracks. (D) Box plot showing average track length. P-values are for an unpaired t test. (E) 100 s time projection of growing microtubules plus-ends marked with EB3-mEGFP after control or GTSE1 RNAi. All scale bars are 10 microns.
(TIF)

Figure S3 **GTSE1 is overexpressed in cancer cell lines and is required for cell migration.** (A)Western blot showing GTSE1 protein levels in transformed and non-transformed cell lines. Non-transformed cell lines are BM (Human bone marrow-derived multipotent adult stem cells), HH (Human heart-derived multipotent adult stem cells), Wi38, and IMR-90. Transformed cell lines are MCF10A, MCF7, MDA-MB-231, HCT116, and U2OS. (B) Western blot showing GTSE1 levels in different cell cycle stages in non-transformed (Wi38) and transformed (U2OS) cell lines. Cells were enriched for cell cycle phases by FACS sorting, and loading was normalized to actin levels. (C) Wound healing migration assay in U2OS cells transfected with a control (CON) or GTSE1 siRNA for 36 hours. Images were taken immediately after cell scraping (0 h) and after 36 hours (36 h).
(TIF)

Figure S4 **GTSE1 expression in breast cancers correlates with clinical outcome and time to metastasis.** (A) Disease Summary for GTSE1: this view displays the number of significant results colored in red or blue for over- or under-expression, respectively, across all cancer types and analysis types in Oncomine. (B) Kaplan–Meier survival curve of breast cancer patients classified according to the expression of GTSE1. Red line: cases with high expression of GTSE1, blue line: cases with low expression of GTSE1. (p<10^∧^−9) (C) Table describing the patients split for the Kaplan–Meier survival curve of time to distant metastasis (TDM) of breast cancer patients classified according to the expression of GTSE1.
(TIF)

Table S1 **Class I phosphorylation sites surrounding SKIP motifs (503–538) in GTSE1 in mitotic HeLa cells.**
(XLSX)

Table S2 **Measurement of cell proliferation by cell counting associated with migration assays.**
(XLSX)

Movie S1 **GTSE1 colocalizes with growing MT plus ends.** U2OS cell stably expressing GTSE1-GFP and mCherry-alpha-tubulin.
(MOV)

Movie S2 **GTSE1 tracks growing MT plus ends in mES cells.** Mouse embryonic stem cell (R1/E) stably expressing GTSE1-GFP.
(MOV)

Movie S3 **GTSE1 tracks growing MT plus ends in U2OS cells.** U2OS GTSE1-GFP cells after CON RNAi.
(MOV)

Movie S4 **GTSE1 does not track growing MT plus ends after EB1 RNAi.** U2OS GTSE1-GFP cells after EB1 RNAi.
(MOV)

Movie S5 **GTSE1-GFP tracks growing MT plus ends.** U2OS cells expressing wild type GTSE1-GFP (GTSE1^WT^_204_)
(MOV)

Movie S6 **Mutations in GTSE1 ‘SKIP’ motifs abolish tracking of MT plus ends**. U2OS cells containing GTSE1-GFP mutated at tandem ‘SKIP’ motifs (GTSE1^Sk^_202_). Mutated GTSE1 does not track growing MT plus ends.
(MOV)

Movie S7 **Purified GTSE1-GFP bind to microtubules.** Purified recombinant hGTSE1-GFP and unlabeled tubulin were added to rhodamine-labeled, GMPCPP stabilized microtubule seeds and imaged by TIRF microscopy.
(MOV)

Movie S8 **GTSE1 microtubule plus-end tracking is downregulated at prometaphase.** U2OS cell stably expressing GTSE1-GFP and EB3-mCherry, stained with Hoechst (DNA). As the cells entered mitosis, short time-lapse movies were acquired every 3 minutes, then stitched together.
(AVI)

Movie S9 **GTSE1 microtubule plus-end tracking resumes at anaphase onset.** U2OS cell stably expressing GTSE1-GFP and EB3-mCherry, stained with Hoechst (DNA). As metaphase cells entered anaphase, short time-lapse movies were acquired every 3 minutes, then stitched together.
(AVI)

Movie S10 **Purified hGTSE1-GFP tracks microtubule plus ends in interphase** ***Xenopus*** **extract.** GMPCPP-stabilized microtubules incubated with interphase *Xenopus* egg extracts and purified hGTSE1-GFP protein imaged by TIRF microscopy.
(MOV)

Movie S11 **Purified hGTSE1-GFP does not track microtubule plus ends in mitotic** ***Xenopus*** **extract.** GMPCPP-stabilized microtubules incubated with mitotic *Xenopus* egg extracts and purified hGTSE1-GFP protein imaged by TIRF microscopy.
(MOV)

Movie S12 **Purified hGTSE1-GFP does not track microtubule plus ends in interphase** ***Xenopus*** **extract after addition of purified CyclinBΔ90.** GMPCPP-stabilized microtubules incubated with interphase *Xenopus* egg extracts, purified hGTSE1-GFP protein, and purified CyclinBΔ90, imaged by TIRF microscopy.
(MOV)

Movie S13 **Purified EB1-GFP tracks microtubule plus ends in interphase** ***Xenopus*** **extract after addition of purified CyclinBΔ90.** GMPCPP-stabilized microtubules incubated with interphase *Xenopus* egg extracts, purified EB1-GFP protein, and purified CyclinBΔ90, imaged by TIRF microscopy.
(MOV)
