# Supplementary figures and images for: GTSE1 Is a Microtubule Plus-End Tracking Protein That Regulates EB1-Dependent Cell Migration
Source: PLoS One. 2012 Dec 7;7(12):e51259. doi: 10.1371/journal.pone.0051259 (PMC3517537; doi:10.1371/journal.pone.0051259)

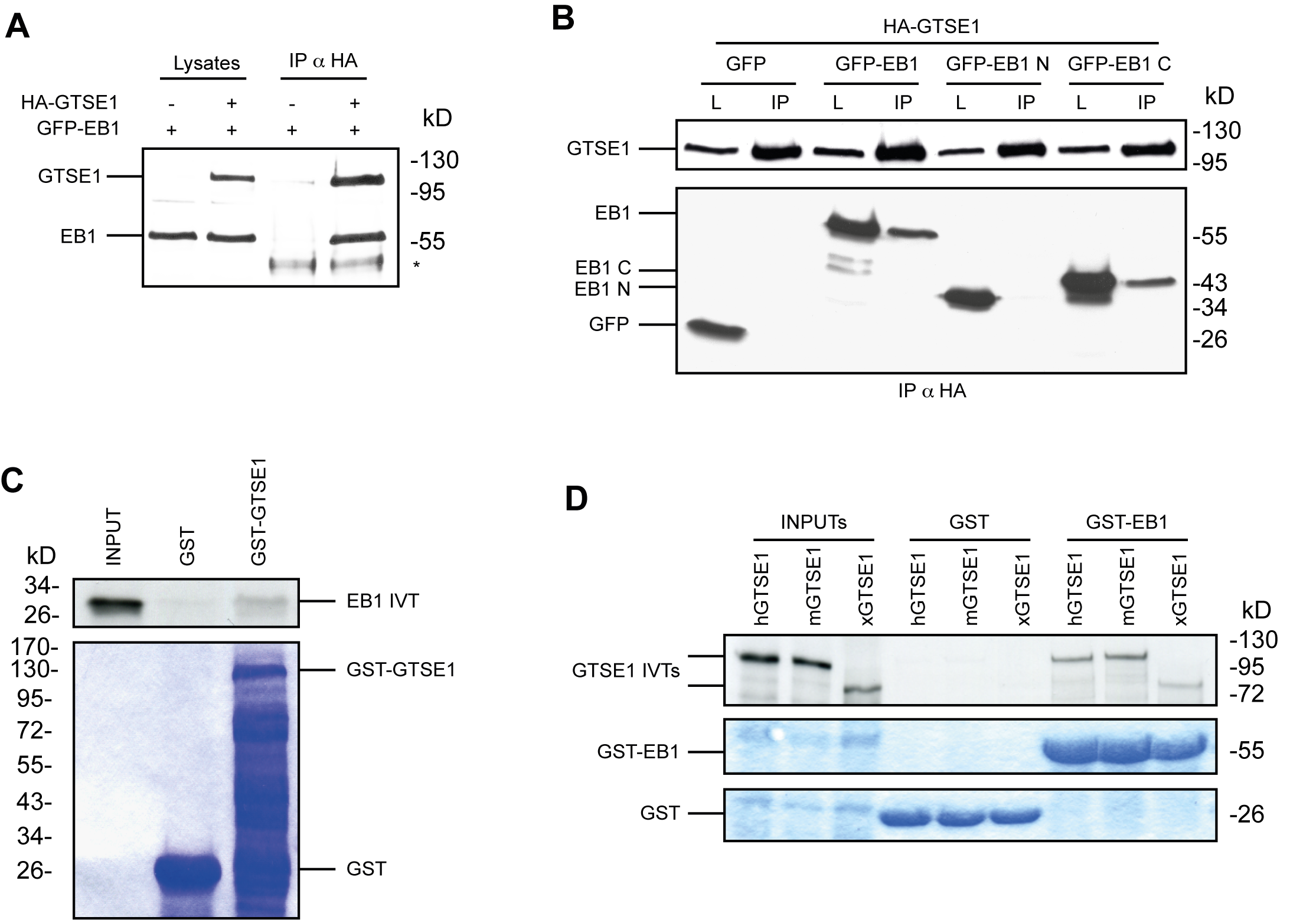

Supplement: Figure S1 — Analysis of GTSE1 interaction with EB1. (A) HEK293T cells were transfected with GFP-tagged EB1 and HA-tagged hGTSE1 for 24 h followed by immunoprecipitation using an anti-HA antibody. Western blots were performed by using anti-HA and -GFP antibodies. * indicates immunoglobulin heavy chain. (B) HEK293T cells were transfected with vectors encoding EB1 deletion mutants (EB1, EB1 N, EB1 C) and HA-hGTSE1, followed by immunoprecipitation using an anti-HA antibody. Anti-GFP and anti-HA antibodies were used for the immunoblot. (C) In vitro pull-down binding assay using recombinant/purified GST and GST-GTSE1 fusion proteins incubated with in vitro translated 35S-labeled EB1 (EB1 IVT). IVT proteins were visualized by autoradiography (Input shows 20% of the EB1 IVT input). Recombinant protein loading was checked by Comassie staining. (D) In vitro pull-down binding assay using recombinant/purified GST and GST-EB1 fusion proteins incubated with in vitro translated 35S-labeled human GTSE1 (hGTSE1), murine GTSE1 (mGTSE1) or Xenopus GTSE1 (xGTSE1). (TIF) [file pone.0051259.s001.tif]

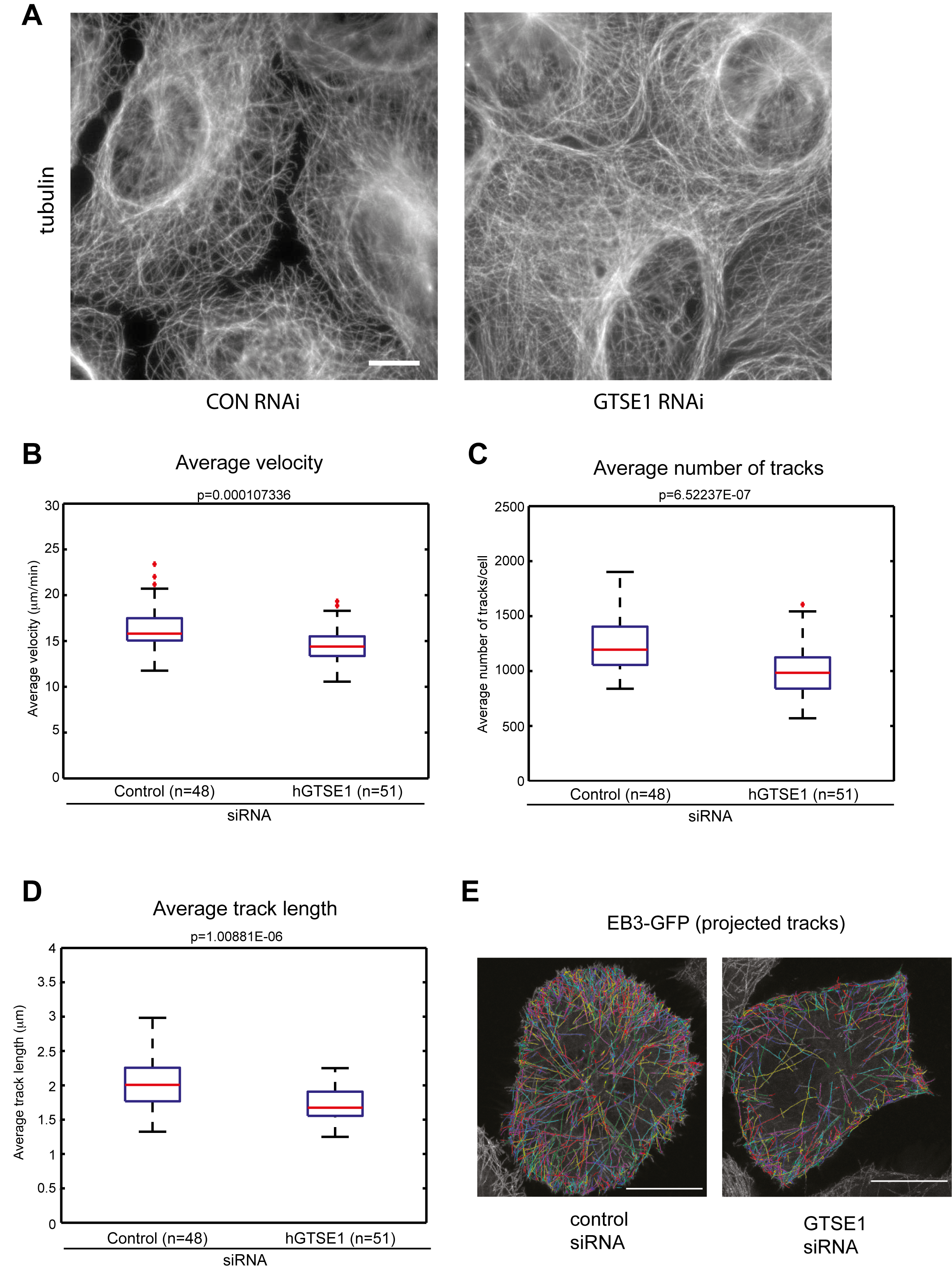

Supplement: Figure S2 — Analysis of MT dynamics after GTSE1 RNAi. (A) Immunofluorescence of U2OS cells transfected with control (CON) or GTSE1 siRNA and stained for alpha-tubulin. (B-E) HeLa cells expressing the plus-end marker EB3-mEGFP were transfected with control or GTSE1 siRNA, and time-lapse imaging performed on interphase cells. (B) Box plot showing EB3 track velocities. (C) Box plot showing number of EB3 tracks. (D) Box plot showing average track length. P-values are for an unpaired t test. (E) 100 s time projection of growing microtubules plus-ends marked with EB3-mEGFP after control or GTSE1 RNAi. All scale bars are 10 microns. (TIF) [file pone.0051259.s002.tif]

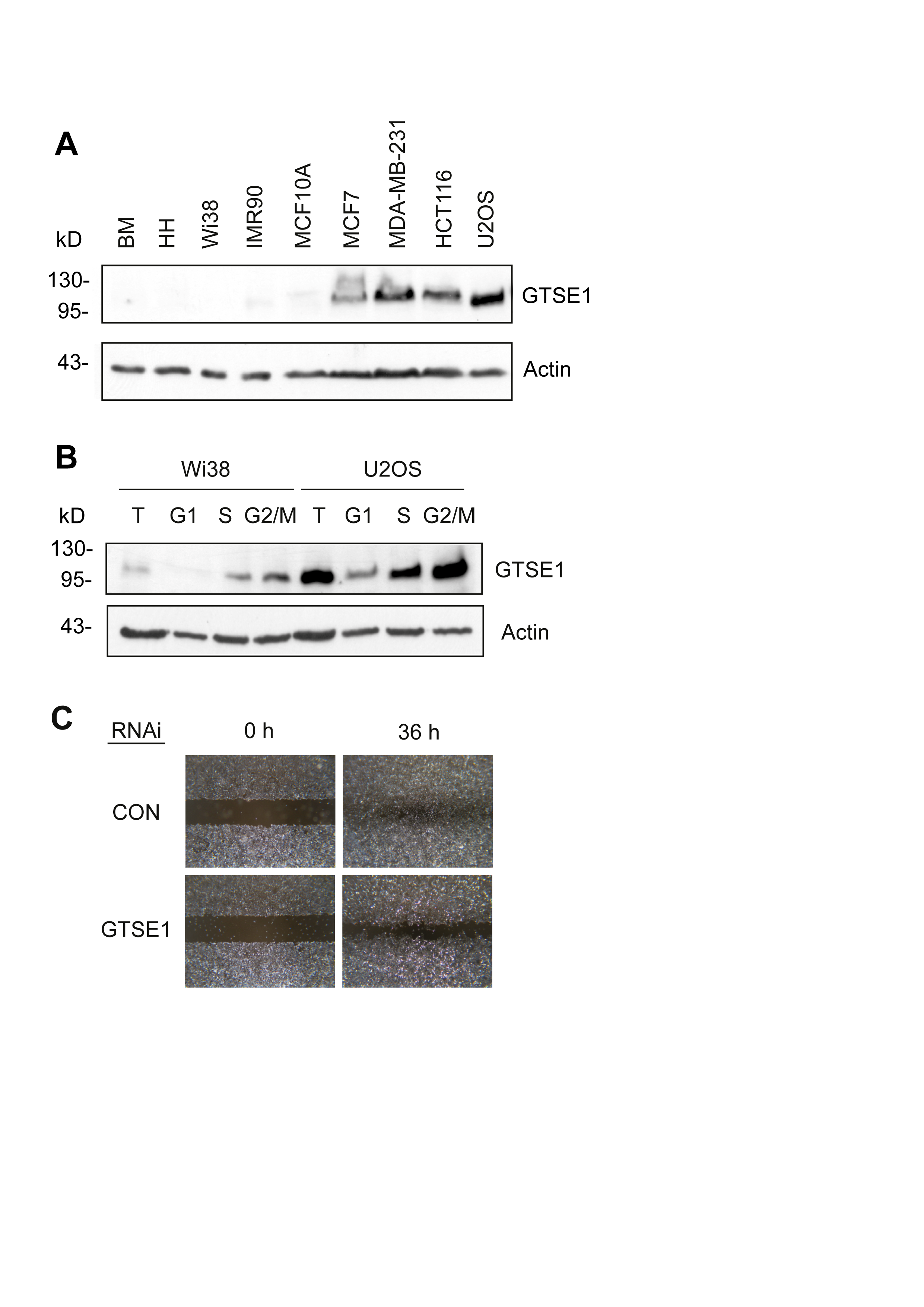

Supplement: Figure S3 — GTSE1 is overexpressed in cancer cell lines and is required for cell migration. (A)Western blot showing GTSE1 protein levels in transformed and non-transformed cell lines. Non-transformed cell lines are BM (Human bone marrow-derived multipotent adult stem cells), HH (Human heart-derived multipotent adult stem cells), Wi38, and IMR-90. Transformed cell lines are MCF10A, MCF7, MDA-MB-231, HCT116, and U2OS. (B) Western blot showing GTSE1 levels in different cell cycle stages in non-transformed (Wi38) and transformed (U2OS) cell lines. Cells were enriched for cell cycle phases by FACS sorting, and loading was normalized to actin levels. (C) Wound healing migration assay in U2OS cells transfected with a control (CON) or GTSE1 siRNA for 36 hours. Images were taken immediately after cell scraping (0 h) and after 36 hours (36 h). (TIF) [file pone.0051259.s003.tif]

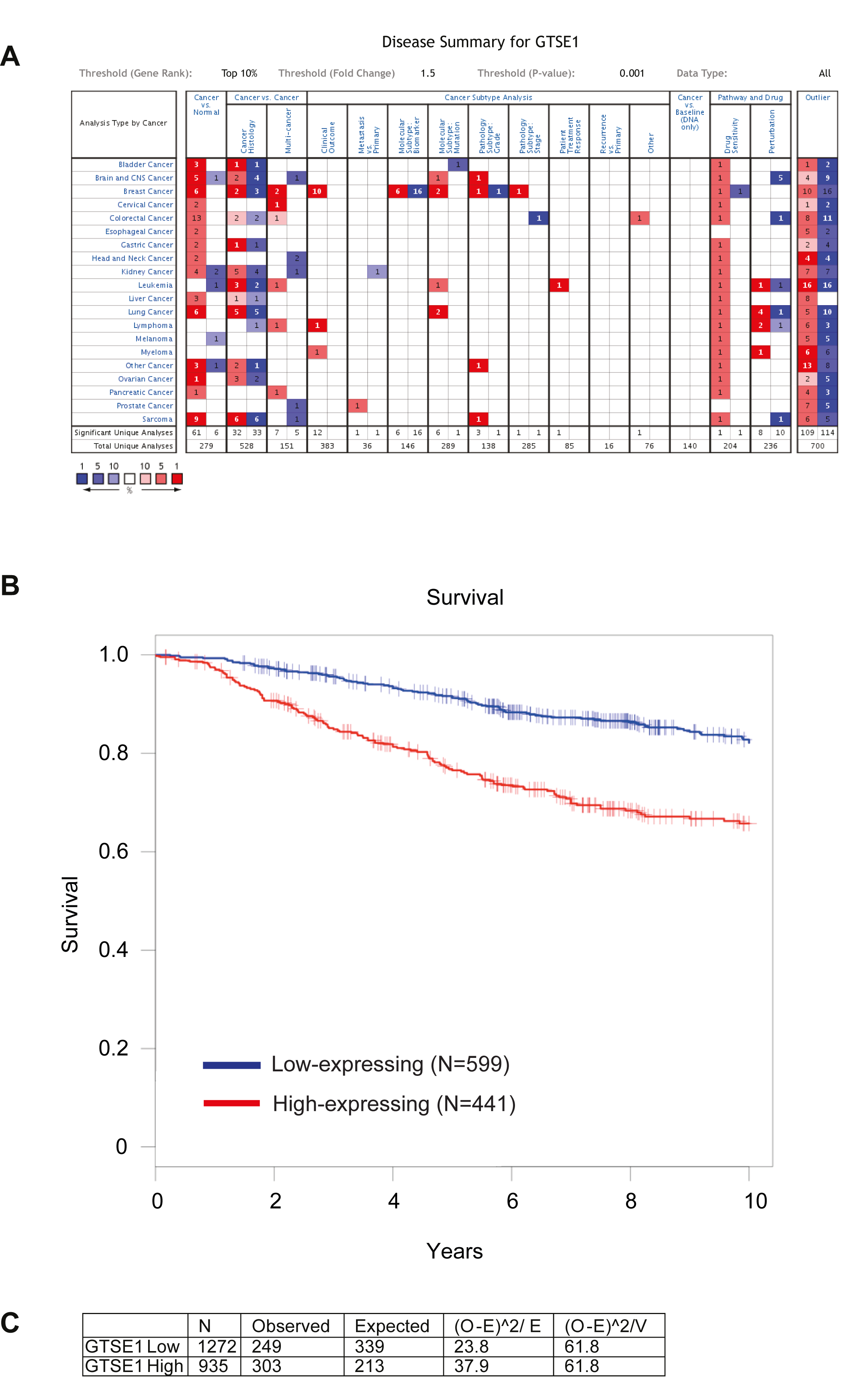

Supplement: Figure S4 — GTSE1 expression in breast cancers correlates with clinical outcome and time to metastasis. (A) Disease Summary for GTSE1: this view displays the number of significant results colored in red or blue for over- or under-expression, respectively, across all cancer types and analysis types in Oncomine. (B) Kaplan–Meier survival curve of breast cancer patients classified according to the expression of GTSE1. Red line: cases with high expression of GTSE1, blue line: cases with low expression of GTSE1. (p<10∧−9) (C) Table describing the patients split for the Kaplan–Meier survival curve of time to distant metastasis (TDM) of breast cancer patients classified according to the expression of GTSE1. (TIF) [file pone.0051259.s004.tif]
